# Off-Label Immunosuppressant Drugs in Solid Organ Transplantation

**DOI:** 10.3390/pharmacy12010017

**Published:** 2024-01-22

**Authors:** Rita Nogueiras-Álvarez, María del Mar García-Sáiz

**Affiliations:** 1 Clinical Trials Unit, Bioaraba Health Research Institute, 01009 Vitoria-Gasteiz, Spain; 2 Clinical Pharmacology Service, Marqués de Valdecilla University Hospital, 39008 Santander, Spain; mmar.garcia@scsalud.es; 3 Department of Physiology and Pharmacology, University of Cantabria, 39012 Santander, Spain; 4 IDIVAL, 39004 Santander, Spain

**Keywords:** off-label prescription, immunosuppressive drugs, immunosuppressants, solid organ transplantation, clinical pharmacology

## Abstract

Once a solid organ transplantation (SOT) has been performed, it is necessary to prescribe immunosuppressant medication to prevent graft rejection. This task has the peculiarity that many of these drugs do not have specific indications for transplant use in the technical data sheets. We performed a review of different immunosuppressive drugs’ information available at European and American regulatory agencies in order to analyze the approved indications by the type of SOT. In our work, besides showing these differences between different indication approvals in different SOT modalities, we also attempted to reflect other differences under the approved indications according to age group, formulation type, geographical area, etc. Although consensus documents on the subject have been published, the access to immunosuppressants depends on each country’s regulation and healthcare system, and off-label prescription is a reality that healthcare professionals need to be familiar with.

## 1. Introduction

Solid organ transplantation (SOT) is a therapeutic option to be considered in many advanced organic diseases. When this option becomes a reality and the transplant candidate is suitable for transplantation, it is then necessary to prescribe post-transplant medication to ensure that the recipient’s immune system does not reject the new organ. The drugs prescribed to prevent rejection in the post-transplant period are the so-called maintenance immunosuppressive drugs.

But an important issue is the lack of standard guidelines for the management of immunosuppression after transplantation.

In fact, there are several differences among the drugs that can be used regarding transplant intrinsic qualities (such as the transplanted organ), but also differences related to each recipients’ characteristics (such as their medical history, age and their tolerability to the medication) or each center’s post-transplant immunosuppression protocols [1].

In addition to the items listed above, there are also a number of prescribing variations of a more bureaucratic nature that are specific for the healthcare region that the patient belongs to. The access to the drugs that are commonly used as immunosuppressive therapy in transplantation is different around the world, and many of them are used off-label, which can make access to medication difficult for many transplant recipients.

**Maintenance immunosuppression therapy after SOT**

It can generally be stated that regardless of the type of organ, the most widely accepted immunosuppressive maintenance therapy used nowadays is a combination of drugs that include a calcineurin inhibitor, an anti-metabolite drug and corticosteroids.

In 2022, Nelson et al. published a consensus with recommendations related to the use of maintenance immunosuppression in SOT [2]. This document reflected, for the different SOT modalities, which drugs were most commonly used as maintenance immunosuppressive therapy.

Table 1 shows the maintenance immunosuppression therapy options reported according to type of transplant [latest data available from the Organ Procurement and Transplantation Network (OPTN) and Scientific Registry of Transplant Recipients (SRTR) 2021 Annual Data Report].

The aim of our study was to review and compare the current status of indications for prescribing different immunosuppressive drugs employed in SOT around the world. For this purpose, we reviewed drugs’ product information and labelling and package inserts.

## 2. Materials and Methods

We performed a review of information available at European and American regulatory agencies about different immunosuppressive drugs used as maintenance therapy after SOT.

The Food and Drug Administration (FDA) website called Drugs@FDA [9] was consulted to look for information about approved therapeutic indications in the United States of America. To find information about the European approved indications, the European Medicines Agency (EMA) website was the source employed [10].

In the case of the European Union, for those drugs that were not authorized using the centralized route, we consulted the technical data sheets on the CIMA website [11] (CIMA is the online drug information center from the Spanish Agency of Medicines and Medical Devices).

In our work, we analyze the approved indications by the type of SOT.

For each immunosuppressant, we have added a brief text with their Anatomical Therapeutic Chemical (ATC) classification. The ATC system [12] is an internationally recognized drug classification system of the World Health Organization that divides the active substances into a hierarchical system on 5 levels according to the organ or system on which they act and their therapeutic, pharmacological and chemical properties. The ATC classification includes the following five different levels:

- ATC 1st level, for anatomical group.
- ATC 2nd level, for therapeutic subgroup.
- ATC 3rd level, for pharmacological subgroup.
- ATC 4th level, for chemical subgroup.
- ATC 5th level identifies the active substance.

## 3. Results: Approved Indications for Different Immunosuppressants Used in SOT

### 3.1. Calcineurin Inhibitors

#### 3.1.1. Tacrolimus

The only tacrolimus formulations used in transplantation are those listed on the ATC classification under the L04AD02 code (which corresponds to the Anatomical Group of antineoplastic and immunomodulatory agents; Therapeutic Group: immunosuppressive agents; Pharmacological Group: immunosuppressants; Chemical Subgroup: calcineurin inhibitors).

Tacrolimus formulations in ointment are excluded as they are not used as immunosuppressants in transplantation and the have another ATC code: D11AH01 (Anatomical Group: dermatological; Therapeutic Group: other dermatological preparations; Pharmacological Group: other dermatological preparations; Chemical Subgroup: dermatitis agents, excluding corticosteroids).

Tacrolimus formulations used as immunosuppressive therapy in transplantation include oral capsules and tablets, sachets and intravenous formulations. All formulations used in transplantation are for hospital prescriptions. The infusion administration option additionally requires that it is used in hospitals.

Tacrolimus-approved indications in SOT:

The therapeutic indications for tacrolimus prolonged-release hard capsules (Advagraf^®^ [13],Tacforius^®^ [14], Astagraf XL^®^ [15,16]) and for the prolonged-release tablets (Envarsus^®^ [17,18], using the MeltDose^®^ delivery technology) include the prophylaxis of organ rejection in adult kidney or liver allograft recipients and the treatment of allograft rejection resistant to treatment with other immunosuppressive medicinal products in adult patients.

The oral hard capsules formulation, the tacrolimus concentrate for solution for infusion (Prograf^®^ hard capsules [19,20], Prograf^®^ concentrate for solution for infusion [20,21]) and the granules for oral suspension (Modigraf^®^ [22], Prograf^®^ [20]) have the following approved indications: prophylaxis of transplant rejection in adult and pediatric, kidney, liver or heart allograft recipients and treatment of allograft rejection resistant to treatment with other immunosuppressants in adult and pediatric patients.

In the USA, tacrolimus updated its approved indications in July 2021 for the following formulations: capsules, injections for intravenous use and granules for oral suspension (Prograf^®^ [23]). This update was based on the results from a non-interventional observational study providing real-world evidence of effectiveness. Since that date, FDA approved tacrolimus for use in combination with other immunosuppressant drugs to prevent organ rejection in adult and pediatric patients receiving lung transplantation [24], as well as the other type of previous transplantation with an indication (liver, kidney and heart). Table 2 summarizes approved indications for tacrolimus in transplantation.

#### 3.1.2. Ciclosporin

The only ciclosporin formulations used in transplantation are those listed on the ATC classification under the L04AD01 code (which corresponds to the Anatomical Group of antineoplastic and immunomodulatory agents; Therapeutic Group: immunosuppressive agents; Pharmacological Group: immunosuppressants; Chemical Subgroup: immunosuppressants).

Ciclosporin formulations in emulsion eye drops are not used as immunosuppressants in transplantation (their ATC code is S01XA18, which corresponds to the Anatomical Group: sensory organs; Therapeutic Group: ophthalmologic agents; Pharmacological Group: other ophthalmologic agents; Chemical Subgroup: other ophthalmologic agents).

Ciclosporin formulations used in SOT include oral soft gelatin capsules, oral solution and concentrate for solution for infusion. As in the case of tacrolimus, the ciclosporin-containing medicines for use in transplantation need a hospital prescription. In addition, the formulations for infusion are only for hospital use.

Ciclosporin-approved indications in transplantation:

In Europe, the ciclosporin technical data sheet includes indications for use in all types of SOT, as prevention of graft rejection and for treatment of cellular rejection (in transplant patients who have previously received other immunosuppressive agents); ciclosporin has indication also in bone marrow transplantation for prevention of graft rejection and for prevention or treatment of graft-versus-host disease (GVHD) (Sandimmun^®^ [25,26], Sandimmun Neoral^®^ [27]).

In the USA, ciclosporin is indicated for the prophylaxis of organ rejection in kidney, liver and heart allogeneic transplants. The technical data sheet (Sandimmune^®^ [28], Neoral^®^ [29]) also states that it is always to be used with adrenal corticosteroids. In addition, ciclosporin may be used for the treatment of chronic rejection in patients previously treated with other immunosuppressive agents. The injection has a warning, with a reminder that this type of formulation should be reserved for patients who are unable to take the capsules or oral solution, as it increases the risk of anaphylaxis.

A summary of approved indications for ciclosporin prescription in SOT can be found in Table 3.

### 3.2. Antimetabolites

#### 3.2.1. Mycophenolic Acid

The ATC classification code for mycophenolic acid is L04AA06 (which corresponds to the Anatomical Group of antineoplastic and immunomodulatory agents; Therapeutic Group: immunosuppressive agents; Pharmacological Group: immunosuppressants; Chemical Subgroup: selective immunosuppressants). Mycophenolic acid is available as two formulations, mycophenolate mofetil (MMP) and mycophenolate sodium (MPS), which are not bioequivalent.

Both MMP and MPS need a hospital prescription.

MMP is available as oral hard capsules, film-coated tablets, powder for oral suspension and powder for concentrate for solution for infusion (CellCept^®^ [30,31]).

MPS is available as delayed-release tablets (Myfortic ^®^ [32,33]). The enteric-coated MPS seems to be a better alternative in the case of gastrointestinal side effects.

Mycophenolic acid-approved indications in transplantation:

On the one hand, MMP formulation in the USA has indication for the prophylaxis of acute transplant rejection in kidney, liver and heart transplantation, whereas in Europe, only oral MMP formulations have an indication for use in these three modalities, while intravenous administration is just indicated for its use in kidney and liver transplantation but not in heart transplantation.

There are also differences in approved MMP indications between Europe and USA depending upon the recipient’s age.

On the other hand, MPS is only approved for prophylaxis of acute rejection in kidney transplant recipients. While in Europe it is approved for use only in adults, when reviewing the USA package insert, we find that it is approved for use both in adults and pediatric recipients above 5 years of age. In any case, if there is a need to prescribe MPS in any SOT other than renal, it would be under an off-label use.

#### 3.2.2. Azathioprine

The ATC classification code for azathioprine is L04AX01 (which corresponds to the Anatomical Group of antineoplastic and immunomodulatory agents; Therapeutic Group: immunosuppressive agents; Pharmacological Group: immunosuppressants; Chemical Subgroup: other immunosuppressants).

Azathioprine-approved indications in transplantation:

Azathioprine is indicated in immunosuppressive regimens as an adjunct to immunosuppressive agents that form the mainstay of treatment (basis immunosuppression).

Azathioprine is available as scored tablets (examples: Imurel^®^ [34], Immufalk^®^ [35], Imuran^®^ [36]) and it can be found also in an oral suspension form.

The tablets are indicated in combination with steroids and/or other immunosuppressive agents to increase the survival in different SOT and to reduce the need for steroids in kidney transplant recipients. The specific indication for each type of SOT should be consulted in the technical data sheet of each product; Imurel^®^, for example, is indicated in kidney, liver and heart transplantation, while Immufalk^®^ product information includes also an indication for lung transplantation.

The product information for the oral suspension form (Jayempi^®^ [37]) states that it is indicated in combination with other immunosuppressive agents (usually corticosteroids) for the prophylaxis of transplant rejection in patients receiving allogenic kidney, liver, heart, lung or pancreas transplants.

Table 4 contains a review of approved indications for antimetabolites in SOT.

### 3.3. m-TOR Inhibitors

#### 3.3.1. Sirolimus

The ATC classification code for sirolimus is L04AA10 (which corresponds to the Anatomical Group of antineoplastic and immunomodulatory agents; Therapeutic Group: immunosuppressive agents; Pharmacological Group: immunosuppressants; Chemical Subgroup: selective immunosuppressants).

Sirolimus-approved indications in transplantation:

Sirolimus (Rapamune^®^ [38,39]) is indicated for the prophylaxis of organ rejection in adult patients at low to moderate immunological risk when receiving a renal transplant. The product information recommends using sirolimus initially in combination with ciclosporin and corticosteroids for 2 to 3 months.

#### 3.3.2. Everolimus

The ATC classification code for everolimus is L04AA18 (which corresponds to the Anatomical Group of antineoplastic and immunomodulatory agents; Therapeutic Group: immunosuppressive agents; Pharmacological Group: immunosuppressants; Chemical Subgroup: selective immunosuppressants).

Everolimus-approved indications in transplantation:

In Europe, everolimus (Certican^®^ [40]) is indicated for the prophylaxis of organ rejection in adult patients who undergo a kidney, liver or heart transplantation. For kidney or heart transplantation, it is only indicated for adult patients at low to moderate immunological risk. These organ receptors should also be treated in combination with ciclosporin and corticosteroids. When everolimus is used for the prophylaxis of organ rejection in liver transplantation, the product information indicates that the m-TOR inhibitor should be prescribed in combination with tacrolimus and corticosteroids.

Similarly, in the USA everolimus (Zortress^®^ [41]) has indication for the prophylaxis of organ rejection in adult patients receiving a kidney transplant if it is to be considered a low to moderate immunologic risk or in liver transplant. Nevertheless, the FDA does not include the indication for heart transplantation.

For a summary of m-TOR inhibitors’ approved indications in SOT, see Table 5.

### 3.4. Corticosteroids

#### 3.4.1. Prednisone

The ATC classification code for prednisone is H02AB07 (which corresponds to the Anatomical Group of systemic hormonal preparations excluding sex hormones and insulins; Therapeutic Group: corticosteroids for systemic use; Pharmacological Group: corticosteroids for systemic use, plain; Chemical Subgroup: glucocorticoids).

Prednisone-approved indications in transplantation:

Prednisone has indication for use in organ transplantation. The product information and package insert do not generally specify the SOT type. The FDA reports that for kidney and heart transplantation, the delayed-release formulation should be specifically prescribed.

#### 3.4.2. Prednisolone

The ATC classification code for prednisolone is H02AB06 (which corresponds to the Anatomical Group of systemic hormonal preparations excluding sex hormones and insulins; Therapeutic Group: corticosteroids for systemic use; Pharmacological Group: corticosteroids for systemic use, plain; Chemical Subgroup: glucocorticoids).

Prednisolone-approved indications in transplantation:

It has indication for use in acute or chronic SOT rejection; although. the product information does not specify the SOT type.

#### 3.4.3. Methylprednisolone

The ATC classification code for methylprednisolone is H02AB04 (which corresponds to the Anatomical Group of systemic hormonal preparations excluding sex hormones and insulins; Therapeutic Group: corticosteroids for systemic use; Pharmacological Group: corticosteroids for systemic use, plain; Chemical Subgroup: glucocorticoids).

Methylprednisolone-approved indications in transplantation:

It could be prescribed for the treatment of acute transplant rejection. Therefore, it is not one of the drugs included in the maintenance immunosuppressive therapy as per se, in contrast with the other drugs listed earlier.

## 4. Discussion

Not all drugs used as immunosuppressive therapy have an indication in all types of SOT. One of the reasons for this is the lack of information from clinical trials demonstrating their efficacy and supporting their use for that particular type of transplantation. Therefore, it is necessary to use them off-label on many occasions [2].

The off-label term refers to any intentional use of an authorized product not covered by the terms of its marketing authorization. Some examples for this include the use of a medicine for an unapproved indication (e.g., life-threatening conditions where physicians may consider that a drug prescription, that is logical and available, is justified regardless of whether it is approved by the regulatory agency for that specific condition or not) and also its use in an unapproved age group, dosage or route of administration [42,43].

Another issue that should be noted is the variability in the access to these drugs depending on the geographic area. In the USA, drug access depends on the approved indications by the FDA and each person’s health insurance provider. This latter factor is a major source of disparity, as many health insurers deny the access to drugs when the indication for which they are prescribed is not listed in the FDA’s label [44,45]. This issue is particularly sensitive in those SOT modalities, such as lung transplantation, which are not listed under the “Indications and Usage” heading of FDA labeling. In this context, pharmacoeconomics studies have been conducted to show the benefits of extending medical coverage in terms of access to medication for transplant recipients in the USA [46].

In our work, we have provided a general overview about the approved indications of different drugs used in immunosuppressive maintenance therapy in SOT.

Previous reports showed quite considerable off-label use of immunosuppressive maintenance medication in SOT receptors, ranging from 16.5% in liver transplant recipients but reaching up to over 65% in lung transplant recipients [44]. Although there is a recent consensus document on the subject [2], the reality is that access to immunosuppressants depends on each country’s regulation and healthcare system. In this way, it becomes clear that many times it is necessary in clinical practice to use an immunosuppressant’s off-label prescription for SOT.

Although different authors have already highlighted these difficulties associated with off-label use of medication in the past [47,48,49], it seems complex to put forward proposals for addressing these differences. When including an indication for the use of a drug in its technical data sheet, the availability of clinical trial results that support its efficacy and safety are important. As previously mentioned, clinical trials in the SOT area are scarce and, as a result, many of the medications that are usually used do not have a specific indication in all SOT modalities. This fact is particularly noteworthy in lung, pancreas or intestine transplants, owing to the smaller number of people receiving these allografts compared to other types of SOT. As an example of this situation during 2022, approximately 6.784 lung transplants were performed, along with 2.026 pancreas transplants and 170 intestine transplants (information of 91 member states on organ transplantation activities, which makes up a 75% of the global population) [50].

The one thing that is fundamental and that becomes evident is that education of healthcare professionals in the off-label use of medication and its implications must be a key element for a rational and responsible drug prescription.

## 5. Conclusions

The off-label use of medication should be taken into account by healthcare professionals with prescriptive competencies. Therefore, medical doctors should become familiar with this type of prescription and be aware of its characteristics.

Those physicians professionally trained in the field of medicines, such as clinical pharmacologists, can be considered as a reference in those situations where different therapeutic alternatives need to be evaluated and when an individualized assessment of a patient’s pharmacological treatment is required, including those where the off-label prescription of a drug is being considered [51].

An example of such situations occurs after SOT, where different immunosuppressive medications are routinely prescribed off-label, as we describe in this text.

## Figures and Tables

**Table 1:** Maintenance immunosuppression therapy in different types of SOT.

Type of SOT	Maintenance Immunosuppression Therapy
Kidney [3]	TAC + corticosteroids + MMP (67.51%) TAC + MMP (25.60%) TAC + corticosteroids (0.71%)
Liver [4]	TAC + corticosteroids + MMP (66.75%) TAC + MMP (17.02%) Other (9.56%) TAC + corticosteroids (5.04%)
Heart [5]	TAC + corticosteroids + MMP (83.93%) TAC + MMP (8.01%) Other (3.20%) TAC + corticosteroids (1.42%)
Lung [6]	TAC + corticosteroids + MMP (81.60%) Other (9.87%) TAC + MMP (2.83%) TAC + corticosteroids (2.83%)
Pancreas [7]	TAC + corticosteroids + MMP (68.15%) TAC + MMP (24.42%) Other (3.18%) TAC + corticosteroids (1.49%)
Intestine [8]	Other (35.42%) TAC + corticosteroids (28.13%) TAC + corticosteroids + MMP (20.83%) TAC + MMP (7.29%)

MMP: mycophenolate mofetil; TAC: tacrolimus.

**Table 2:** Approved indications for tacrolimus in transplantation.

	IR-Tac Hard Capsules (Prograf^®^-Europe, USA)	PR-Tac Prolonged-Release Hard Capsules (Advagraf^®^-Europe, Tacforius^®^-Europe, Astagraf XL^®^-USA)	LCP-Tac Prolonged-Release Tablets (Envarsus XR^®^-Europe, USA)	Tac Granules for Oral Suspension (Modigraf^®^-Europe, Prograf^®^-USA)	Tac Concentrate for Solution for Infusion (Prograf^®^-Europe, USA)
**Kidney**	Europe: 1 (adult, pediatric), 4 (adult, pediatric) USA: 1 (adult, pediatric)	Europe: 1 (adult), 2 (adult) USA: 1 (*)	Europe: 1 (adult), 2 (adult) USA: 1 (*), 3 (*)	Europe: 1 (adult, pediatric), 4 (adult, pediatric) USA: 1 (adult, pediatric)	Europe: 1 (adult, pediatric), 4 (adult, pediatric) USA: 1 (adult, pediatric)
**Liver**	Europe: 1 (adult, pediatric) USA: 1 (adult, pediatric)	Europe: 1 (adult), 2 (adult) USA: OFF-LABEL	Europe: 1 (adult), 2 (adult) USA: OFF-LABEL	Europe: 1 (adult, pediatric), 4 (adult, pediatric) USA: 1 (adult, pediatric)	Europe: 1 (adult, pediatric), 4 (adult, pediatric) USA: 1 (adult, pediatric)
**Heart**	Europe: 1 (adult, pediatric) USA: 1 (adult, pediatric)	Europe: 2 (adult) USA: OFF-LABEL	Europe: OFF-LABEL USA: OFF-LABEL	Europe: 1 (adult, pediatric), 4 (adult, pediatric) USA: 1 (adult, pediatric)	Europe: 1 (adult, pediatric), 4 (adult, pediatric) USA: 1 (adult, pediatric)
**Lung**	Europe: ^ OFF-LABEL USA: 1 (adult, pediatric)	Europe: OFF-LABEL USA: OFF-LABEL	Europe: OFF-LABEL USA: OFF-LABEL	Europe: ^ OFF-LABEL USA: 1 (adult, pediatric)	Europe: ^ OFF-LABEL USA: 1 (adult, pediatric)
**Pancreas**	Europe: ^ OFF-LABEL USA: OFF-LABEL	Europe: OFF-LABEL USA: OFF-LABEL	Europe: OFF-LABEL USA: OFF-LABEL	Europe: ^ OFF-LABEL USA: OFF-LABEL	Europe: ^ OFF-LABEL USA: OFF-LABEL
**Intestine**	Europe: ^ OFF-LABEL USA: OFF-LABEL	Europe: OFF-LABEL USA: OFF-LABEL	Europe: OFF-LABEL USA: OFF-LABEL	Europe: ^ OFF-LABEL USA: OFF-LABEL	Europe: ^ OFF-LABEL USA: OFF-LABEL

IR-Tac: immediate-release tacrolimus; LCP-Tac: new prolonged-release formulation of tacrolimus; PR-Tac: prolonged-release tacrolimus; Tac: tacrolimus; USA: United States of America. * Age group not specified. 1: Prophylaxis of transplant rejection in allograft recipients. 2: Treatment of allograft rejection after transplantation in adult patients. 3: Prophylaxis of organ rejection in kidney transplant patients converted from tacrolimus immediate-release formulations in combination with other Immunosuppressants. 4: Treatment of allograft rejection resistant to treatment with other immunosuppressive drugs. ^: Treatment of allograft rejection after transplantation of other allografts—dose recommendations for lung, pancreas and intestine transplantation are based on limited data from prospective clinical trials with the Prograf^®^ formulation.

**Table 3:** Approved indications for ciclosporin in transplantation.

	Cs Oral Soft Gelatin Capsules (Sandimmun^®^-Europe, Sandimmun Neoral^®^-Europe, Sandimmune^®^-USA, Neoral^®^-USA)	Cs Oral Solution (Sandimmun^®^-Europe, Sandimmun Neoral^®^-Europe, Sandimmune^®^-USA, Neoral^®^-USA)	Cs Injection Concentrate for Solution for Infusion (Sandimmun^®^-Europe, Sandimmune^®^-USA, Neoral^®^-USA)
**Kidney**	Europe: 1, 2 (*) USA: 1 [Sandimmune^®^ also for 3] (*)	Europe: 1, 2 (*) USA: 1 [Sandimmune^®^ also for 3] (*)	Europe: 1, 2 (*) USA: 1 [Sandimmune^®^ also for 3] (*)
**Liver**	Europe: 1, 2 (*) USA: 1 [Sandimmune^®^ also for 3] (*)	Europe: 1, 2 (*) USA: 1 [Sandimmune^®^ also for 3] (*)	Europe: 1, 2 (*) USA: 1 [Sandimmune^®^ also for 3] (*)
**Heart**	Europe: 1, 2 (*) USA: 1 [Sandimmune^®^ also for 3] (*)	Europe: 1, 2 (*) USA: 1 [Sandimmune^®^ also for 3] (*)	Europe: 1, 2 (*) USA: 1 [Sandimmune^®^ also for 3] (*)
**Lung**	Europe: 1, 2 (*) USA: OFF-LABEL	Europe: 1, 2 (*) USA: OFF-LABEL	Europe: 1, 2 (*) USA: OFF-LABEL
**Pancreas**	Europe: 1, 2 (*) USA: OFF-LABEL	Europe: 1, 2 (*) USA: OFF-LABEL	Europe: 1, 2 (*) USA: OFF-LABEL
**Intestine**	Europe: 1, 2 (*)	Europe: 1, 2 (*)	Europe: 1, 2 (*)

Cs: ciclosporin. * Age group not specified. 1: Prevention of graft rejection following solid organ transplantation. 2: Treatment of transplant cellular rejection in patients previously receiving other immunosuppressive agents. 3: Treatment of chronic rejection in patients previously treated with other immunosuppressive agents.

**Table 4:** Approved indications for antimetabolites in transplantation.

	MPA					Azathioprine	
	MMP				MPS		
	Hard Capsules (CellCept^®^-Europe, USA)	Film-Coated Tablets (CellCept^®^-Europe, USA)	Powder for Oral Suspension (CellCept^®^-Europe, USA)	Powder for Concentrate for Solution for Infusion (CellCept^®^-Europe, USA)	Delayed-Release Tablets (Myfortic^®^-Europe, USA)	Scored Tablets (Imurel^®^-Europe, Immufalk^®^-Europe, Imuran^®^-Europe, USA)	Oral Suspension (Jayempi^®^-Europe)
Kidney	Europe: 1 (adult, pediatric) USA: 2 (adult, pediatric recipients ≥3 months of age)	Europe: 1 (adult, pediatric) USA: 2 (adult, pediatric recipients ≥3 months of age)	Europe: 1 (adult, pediatric) USA: 2 (adult, pediatric recipients ≥3 months of age)	Europe: 1 (adult) USA: 2 (adult)	Europe: 1 (adult) USA: 1 (adult, pediatric recipients ≥5 years of age who are at least 6 months post kidney transplant)	Europe: 2 (*) USA: 3 (*)	Europe: 2 (*)
Liver	Europe: 1 (*) USA: 2 (adult, pediatric recipients ≥3 months of age)	Europe: 1 (*) USA: 2 (adult, pediatric recipients ≥3 months of age)	Europe: 1 (*) USA: 2 (adult, pediatric recipients ≥3 months of age)	Europe: 1 (adult) USA: 2 (adult)	Europe: OFF-LABEL USA: OFF-LABEL	Europe: 2 (*) USA: OFF-LABEL	Europe: 2 (*) USA: OFF-LABEL
Heart	Europe: 1 (*) USA: 2 (adult, pediatric recipients ≥3 months of age)	Europe: 1 (*) USA: 2 (adult, pediatric recipients ≥3 months of age)	Europe: 1 (*) USA: 2 (adult, pediatric recipients ≥3 months of age)	Europe: OFF-LABEL USA: 2 (adult)	Europe: OFF-LABEL USA: OFF-LABEL	Europe: 2 (*) USA: OFF-LABEL	Europe: 2 (*) USA: OFF-LABEL
Lung	Europe: OFF-LABEL USA: OFF-LABEL	Europe: OFF-LABEL USA: OFF-LABEL	Europe: OFF-LABEL USA: OFF-LABEL	Europe: OFF-LABEL USA: OFF-LABEL	Europe: OFF-LABEL USA: OFF-LABEL	Europe: 2 (*) USA: OFF-LABEL	Europe: 2 (*) USA: OFF-LABEL
Pancreas	Europe: OFF-LABEL USA: OFF-LABEL	Europe: OFF-LABEL USA: OFF-LABEL	Europe: OFF-LABEL USA: OFF-LABEL	Europe: OFF-LABEL USA: OFF-LABEL	Europe: OFF-LABEL USA: OFF-LABEL	Europe: OFF-LABEL USA: OFF-LABEL	Europe: 2 (*) USA: OFF-LABEL

MPA: mycophenolic acid; MMP: mycophenolate mophetil; MPS: mycophenolate sodium. * Age group not specified. 1: In combination with ciclosporin and corticosteroids, it is indicated for the prophylaxis of acute transplant rejection. 2: Prophylaxis of organ rejection in combination with other immunosuppressants. 3: As an adjunct for the prevention of rejection in renal homotransplantation.

**Table 5:** Approved indications for mTOR inhibitors in transplantation.

	Sirolimus		Everolimus
	Coated Tablets (Rapamune^®^-Europe, USA)	Oral Solution (Rapamune^®^-Europe, USA)	Tablets (Certican^®^-Europe, Zortress^®^-USA)
Kidney	Europe: 1 (adult) USA: 1 (adult, pediatric recipients ≥13 years of age)	Europe: 1 (adult) USA: 1 (adult, pediatric recipients ≥13 years of age)	Europe: 1 (adult patients at low to moderate immunological risk) USA: 1 (adult)
Liver	No data	No data	Europe: 1 (adult) USA: 1 (adult)
Heart	No data	No data	Europe: 1 (adult patients at low to moderate immunological risk)

1: Prophylaxis of acute transplant rejection in combination with other immunosuppressants.

## Data Availability

All data analyzed during this study are included in this article.
